# Peritoneal carcinomatosis management through cytoreductive surgery with hyperthermic intraperitoneal chemotherapy: first experience in Iraq

**DOI:** 10.3389/fonc.2025.1667950

**Published:** 2026-01-16

**Authors:** Aqeel Shakir Mahmood, Ahmed Dheyaa Al-Obaidi, Mustafa Najah Al-Obaidi, Yousif Ali Madlul, Ahmed Sermed Al Sakini, Hashim Talib Hashim, Ahmed A. Shakir, Tabarak Qassim, Aya Ahmed Shimal, Marafi Jammaa Ahmed, Fatima Elbasri Abuelgasim Mohammed, Hasan Al-Obaidi

**Affiliations:** 1 College of Medicine Baghdad, Baghdad University, Baghdad, Iraq; 2 Chairman of the Scientific Council of Gastrointestinal Surgery at the Iraqi Council for Medical Specialties, Baghdad, Iraq; 3 Bachelor of Medicine, Bachelor of Surgery (MBChB), College of Medicine, University of Baghdad, Baghdad, Iraq; 4 College of Medicine, University of Baghdad, Baghdad, Iraq; 5 College of Medicine, University of Warith Al-Anbiyaa, Karbala, Iraq; 6 General Surgery Department, Medical City Complex, Baghdad, Iraq; 7 General Surgery Department, Royal College of Surgeons in Ireland-Bahrain, Muharraq, Bahrain; 8 General Surgery Department, Faculty of Medicine, Bahri University, Khartoum, Sudan; 9 Faculty of Medicine, Khartoum University, Khartoum, Sudan; 10 Doctor of Medicine (MD), Jamaica Hospital Medical Center, New York, NY, United States

**Keywords:** CRS, cytoreductive surgery, HIPEC, hyperthermic intraperitoneal chemotherapy, neoadjuvant chemotherapy, peritoneal carcinomatosis

## Abstract

**Background:**

Given the current lack of data from Iraq regarding peritoneal carcinomatosis (PC) management using Cytoreductive Surgery (CRS) with Hyperthermic Intraperitoneal Chemotherapy (HIPEC), and the diversity in genetics, environmental conditions, and socioeconomic factors in Iraq, we conducted the first study of PC management using CRS+HIPEC.

**Methods:**

This is a retrospective single-center study at Al-Arabi Private Hospital in Baghdad, Iraq, on 61 patients recently diagnosed with peritoneal cancer who have been managed by CRS and HIPEC for the first time in Iraq. Subsequently, the patients were observed for a duration of 4 years throughout the follow-up phase.

**Results:**

The study, with an average participant age of 49.15 years and females representing 62%, showed an overall survival rate of 26.2% and a median survival duration of 32.51+/-12.8 months. Colorectal and ovarian cancers were predominant at 49% and 21%, respectively. The closed method of HIPEC was used in 80% of cases. Significant associations were found between Neoadjuvant Chemotherapy (NACT) (p = 0.0001), Cytoreductive Surgery score (CC score) (p = 0.01), Peritoneal Cancer Index (PCI) (p = 0.001), and survival rates.

**Conclusion:**

In summary, as Iraq’s first study, our study provides crucial insights into PC management. Highlighting the vital role of NACT in CRT+HIPEC treatment, our study reveals no statistically significant difference between closed and open HIPEC methods. Our study further emphasizes the prognostic value of an elevated PCI and the critical importance of optimal cytoreduction, as reflected in the Completeness of Cytoreduction (CC) score. Our findings call for continued research, collaboration, and practical application to advance PC care.

## Introduction

Peritoneal carcinomatosis (PC) is a relatively rare form of cancer that affects the serous membrane encasing the abdominal cavity. Peritoneal cancer mainly arises from a sequence of mutations occurring in the peritoneal cells. Alternatively, it might be a secondary tumor originating from other organs such as the stomach, colon, pancreas, gallbladder, appendix, breast, uterus, ovary, and lungs. The primary cancer is classified as stage III or IV, whereas metastasis is considered as stage IV (1).

Due to the limited effectiveness of systematic chemotherapy in treating peritoneal cancer (PC), a novel technique of cytoreductive surgery (CRS) and hyperthermic intraperitoneal chemotherapy (HIPEC) has emerged as an alternative treatment option (2). Furthermore, this surgery may serve as a preventive measure against peritoneal metastases arising from the stomach or colon, in addition to its role in treating PC (3, 4).

Numerous studies have consistently shown that the combination of both CRS and HIPEC leads to a significant improvement in survival rates (5–8). Both our study and earlier studies have verified that administering neoadjuvant chemotherapy (NACT) before starting CRS with HIPEC offers significant advantages over doing CRS or HIPEC alone (9, 10).

The procedure involves resecting out all visible macroscopic tumors to a minimum. A complete CRS with no visible tumor is desirable. HIPEC then targets the remaining tiny cancer cells. The peritoneum is infused with a heated (42°C) chemotherapeutic treatment for 60–120 minutes. Mitomycin C, irinotecan, doxorubicin, oxaliplatin, and cisplatin are the most common chemotherapeutic drugs (2, 11).

The HIPEC procedure may be conducted using either the closed abdomen technique or the open technique. The open approach involves creating a funnel shape by opening and elevating the abdominal wall. The chemotherapeutic drug flows via artificial tubes surrounding the funnel. The tubes are connected to a pump and heater. The heated chemotherapeutic drug is distributed evenly in the abdominal cavity using this method. With this method, surgeons may be certain that the anastomosis will remain intact since it is performed after HIPEC. However, since the abdomen is open during perfusion, transpiration of heat occurs, making hyperthermic conditions difficult to reach. Personnel exposure to cytotoxic chemotherapy agents is also possible.

However, the closed approach inserts tubes via abdominal wall incisions. After abdominal wall closure, HIPEC is administered. In contrast to the open approach, the chemotherapeutic drug is distributed unevenly. A disparate distribution of HIPEC raises a pair of challenges. One issue is that the heated agent fails to penetrate all portions of the abdomen, which might reduce the effectiveness of the CRS with the HIPEC operation. The second concern is that the heated agents might be applied too heavily to some places inside the abdomen, leading to hyperthermic damage. Complications include postoperative ileus, intestinal perforation, and fistulas, which are more common as a consequence. One advantage of this method is that it mayquickly reach a hyperthermic condition since very little heat is dissipated. Moreover, it protects the surgical team from the cytotoxic agents.

This procedure involves monitoring the patient’s temperature using an esophageal probe. It is anticipated for the patient’s temperature to climb to 39 degrees Celsius or higher. Cooling procedures, such as placing him on a special cooling blanket, would be performed to ensure their body was maintained at a safe temperature. Intravenous cold crystalloids or head and neck ice packs are further options (11–13).

The objective of this research is to fill the existing lack of data on peritoneal carcinomatosis (PC) care using cytoreductive surgery (CRS) and hyperthermic intraperitoneal chemotherapy (HIPEC) in Iraq. Leveraging the distinctive genetics and demographics of Iraqis, our research seeks to augment existing literature. Examining the impact of CRS+HIPEC on Iraqi patients with PC, this study represents the pioneering implementation of this treatment in Iraq. Our results provide a novel perspective on the effectiveness of CRS+HIPEC in a developing country setting by combining unique data and contributing valuable insights to the medical community.

## Methods

### Patients’ selection and settings

A single-center study from Al-Arabi Private Hospital in Baghdad, Iraq conducted a retrospective analysis of 61 patients with newly diagnosed peritoneal cancer, who were treated with CRS and HIPEC for the first time in Iraq between 13th January 2019 and 26th October 2019. During the initial screening phase, a total of 66 patients were assessed for eligibility; 5 patients were excluded (3 patients refused HIPEC and 2 patients had extraperitoneal metastasis), resulting in a final study population of 61 patients. Subsequently, the patients were observed for a duration of 4 years of follow-up (median follow-up: 48 months; range: 6–48 months), which ended on October 26th, 2023.

A variety of diagnostic tools were employed for the purpose of patient diagnosis and selection. These techniques include contrast-enhanced computer tomography (CT), magnetic resonance imaging (MRI), positron emission tomography (PET), serum tumor markers, diagnostic laparoscopy, and the consideration of center features such as experience, certification, and register study. Several aspects of the tumor itself including the initial site, Peritoneal Cancer Index (PCI), histology, and chemosensitivity, in addition to patients’ characteristics, were of particular importance in determining eligibility for surgery.

All included patients completed the planned follow-up period, and no patients were lost to follow-up during the study duration.

- Inclusion Criteria.
  1. Definitive diagnosis of peritoneal carcinomatosis.
  2. Ability to undergo HIPEC based on multidisciplinary team decision and medical qualification.
  3. Cancer is resectable.
  4. PCI < 25.
- Exclusion Criteria.
  1. Extraperitoneal metastasis.
  2. Unresectable tumor.
  3. The patient isn’t eligible for undergoing surgery based on a multidisciplinary team decision.
  4. Patient’s preference for palliation or HIPEC refusal.

A total of 5 patients were excluded based on these criteria: 3 patients declined HIPEC treatment, and 2 patients were found to have extraperitoneal metastasis.

### Data collection

Data collection was handled through the utilization of a specialized case report form (CRF) that was particularly designed for the study. The collected data was then recorded in the hospital database, ensuring complete privacy. Any additional required data were obtained by in-person appointments.

The demographic, clinical, and surgical variables of each patient were examined about both overall postoperative morbidity and mortality. The study examined various characteristics including age, body mass index (BMI), American Society of Anesthesiologist (ASA) score, Eastern Cooperative Group (ECOG) score, presence of ascites, NACT within 60 days, HIPEC technique, PCI, number of blood transfusions, operative time, and Completeness of Cytoreduction Score (CC) score.

### HIPEC surgical techniques, and chemotherapy

Regarding HIPEC techniques, the closed abdomen or coliseum technique was applied in our center. Once the cancer had been surgically resected, perfusion was performed via open or closed abdominal surgery by inserting catheters and suction drains through the abdominal wall. The procedure was conducted under hyperthermic conditions due to its direct cytotoxic impact and its ability to enhance the efficacy and depth of penetration of chemotherapeutic agents.

A thermal exchange apparatus maintained the injected fluid at 46–48°C, keeping the intraperitoneal fluid at 41–43°C for a total duration ranging from 30 to 120 minutes. This variation was protocol-driven and depended on the chemotherapeutic regimen administered and the primary tumor origin, rather than operator preference. Oxaliplatin-containing regimens were delivered over shorter durations (30 minutes), whereas mitomycin C–based regimens and cisplatin–paclitaxel combinations were administered over longer durations (90–120 minutes) (14, 15).

Regarding chemotherapeutic agents used for peritoneal carcinomatosis during our HIPEC procedure, mitomycin C and oxaliplatin were administered as combined agents for patients with primary colorectal, gastric, and appendiceal cancers (16). A combination of cisplatin and paclitaxel was administered for patients diagnosed with primary ovarian cancer (17).

### Ethical considerations

All patients were informed of the HIPEC goals, potential outcomes, and surgical risks. Each of them provided written consent after being given a thorough explanation of the surgical procedure and chemotherapy.

Patients were informed that their information would be used for research purposes. All procedures conducted adhered to the ethical criteria set by the center researchcommittee and were in compliance with the principles outlined in the 1964 Helsinki Declaration.

### Statistical analysis

The study included calculation of post-HIPEC overall survival (OS) and evaluation of factors affecting mortality and postoperative complications over a 4-year follow-up period. Survival rates were determined using the Kaplan–Meier method. Overall survival, survival stratified by NACT, PCI, CC score, and surgical technique were analyzed using Kaplan–Meier survival analysis.

A non-parametric approach was employed due to the presence of atypical distributions in certain numerical variables and the relatively small sample size. Statistical analysis was performed using the Statistical Package for Social Sciences (SPSS), version 25 (SPSS Inc., Chicago, Illinois, USA). Quantitative variables were presented as mean ± standard deviation (SD), while categorical variables were expressed as frequencies and percentages. A p-value < 0.05 was considered statistically significant.

## Results

### Patient demographics and baseline characteristics

The mean age of the study population was 49.15 ± 9.9 years. The mean body mass index (BMI) was 27.6 ± 4.3 kg/m². Females represented 62% of the population, while males represented 38%. At the end of the 4-year follow-up period, 74% of the participants had died. The median survival duration for the entire group was 32.51 months, with a standard deviation of 12.8 months. Among the participants, 67% had a PCI of 20 or less, with an average value of 19.82 and a standard deviation of 4.9. The cytoreductive surgery score had an average value of 1.5 with a standard deviation of 0.906. The predominant primary tumors seen in our study were colorectal and ovarian cancer (49% and 21%, respectively). The average Peritoneal Cancer Index (PCI) was 19.82 ± 4.9, reflecting a high tumor burden at presentation. The mean Completeness of Cytoreduction (CC) score was 1.5 ± 0.91. Operative procedures were extensive, with a mean operative time of 528 ± 62.6 minutes. Postoperatively, patients required a mean intensive care unit stay of 2.48 ± 1.01 days. Patient demographic and baseline characteristics are summarized in **Table 1**.

**Table 1:** Patient characteristics, operative variables, and factors associated with overall survival.

Variables		Mortality		P value
		Alive	Dead	
Sex	Female	10	28	0.984
	Male	6	17	
primary ca.	Colorectal	8	22	0.865
	Gastric	2	6	
	Ovarian	3	10	
	Appendiceal	1	1	
	Peritoneal	2	6	
Ascites	None	10	33	0.414
	Present	6	12	
Eastern Cooperative Oncology Group	0	3	9	0.949
	1	5	14	
	2	6	14	
	3	2	8	
American Society of Anesthesiologists	1	4	10	0.952
	2	7	22	
	3	4	9	
	4	1	4	
Technique of HIPEC	closed	14	35	0.401
	Open	2	10	
Resected organs	1	0	4	0.786
	2	8	20	
	3	2	7	
	4	3	7	
	5	3	7	
Number of blood transfusions	0	0	4	0.534
	1	7	16	
	2	7	22	
	3	2	3	
Neoadjuvant Chemotherapy	No	1	30	0.0001
	Yes	15	15	
Cytoreductive Surgery score	0	3	6	0.01
	1	10	10	
	2	3	21	
	3	0	8	
Peritoneal Cancer score	>20	0	20	0.001
	≤20	16	25	
anemia	No	16	43	0.391
	Yes	0	2	
Nausea and vomiting	No	16	37	0.07
	Yes	0	8	
Pleural effusion	No	15	41	0.741
	Yes	1	4	
pancreatitis	No	15	41	0.741
	Yes	1	4	
sepsis	No	16	43	0.391
	Yes	0	2	
Intestinal perforation	No	16	43	0.391
	Yes	0	2	
Age		50.81+/-10.12	48.56+/-9.9	0.76
Body Mass Index		23.7+/-3.7	25.13+/-43	0.231
Median survival duration (mean +/- SD)		48	27+/-10.353	0.0001

### Perioperative morbidity and complications

The closed HIPEC technique was the most frequently used approach, accounting for 80% of cases, while the open technique was applied in 20%. The mean operative time was 528 ± 62.59 minutes. Multiple organ resections were commonly required, with 46% of patients undergoing resection of two organs and 47% undergoing resection of three or more organs. Blood transfusions were administered in the majority of patients, with 86% receiving one or more units. The mean intensive care unit stay was 2.48 ± 1.01 days.

Perioperative morbidity was observed in a subset of patients undergoing CRS and HIPEC. According to the Clavien–Dindo classification, the majority of postoperative complications were minor (grades I–II), including nausea and vomiting (13%), pleural effusion (8%), pancreatitis (8%), and anemia (3%), all of which were managed conservatively. Major complications (grades III–IV) were infrequent and included intestinal perforation (3%) and sepsis (3%), both requiring invasive intervention. No Clavien–Dindo grade V (perioperative mortality) events were recorded (**Table 1**).

### Factors associated with survival

Correlation analysis demonstrated statistically significant associations between survival and neoadjuvant chemotherapy (p = 0.0001), Completeness of Cytoreduction score (p = 0.01), and Peritoneal Cancer Index (p = 0.001). Other demographic, clinical, and operative variables did not show statistically significant associations with survival. These results are summarized in **Table 1**.

### Survival outcomes

**Figure 1:**
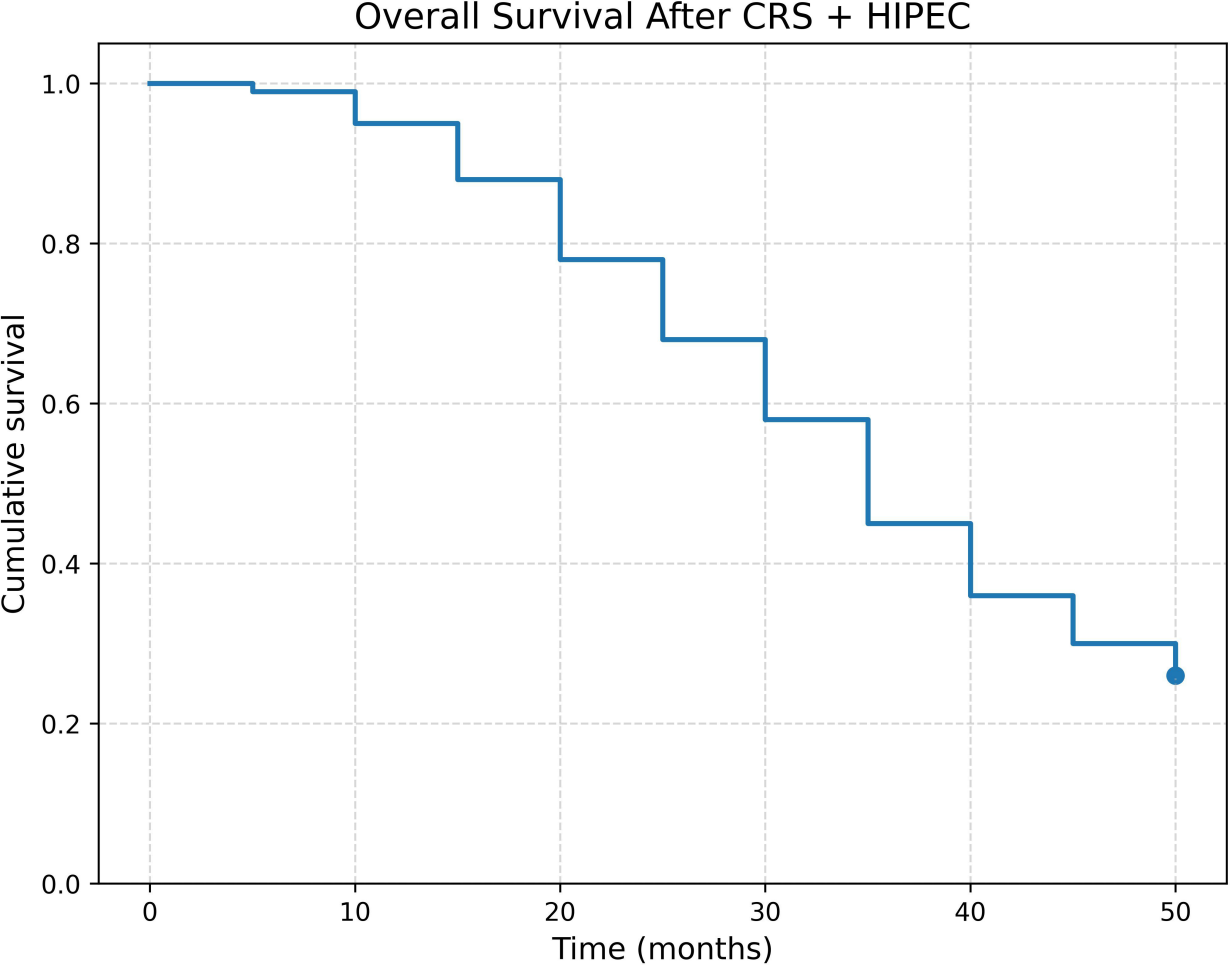
Overall survival curve of patients underwent CRS + HIPEC.

The study revealed an overall survival (OS) rate of 26.2%, accompanied by a median survival duration of 32.51 ± 12.8 months (**Figure 1**).

### Impact of neoadjuvant chemotherapy on survival.

**Figure 2:**
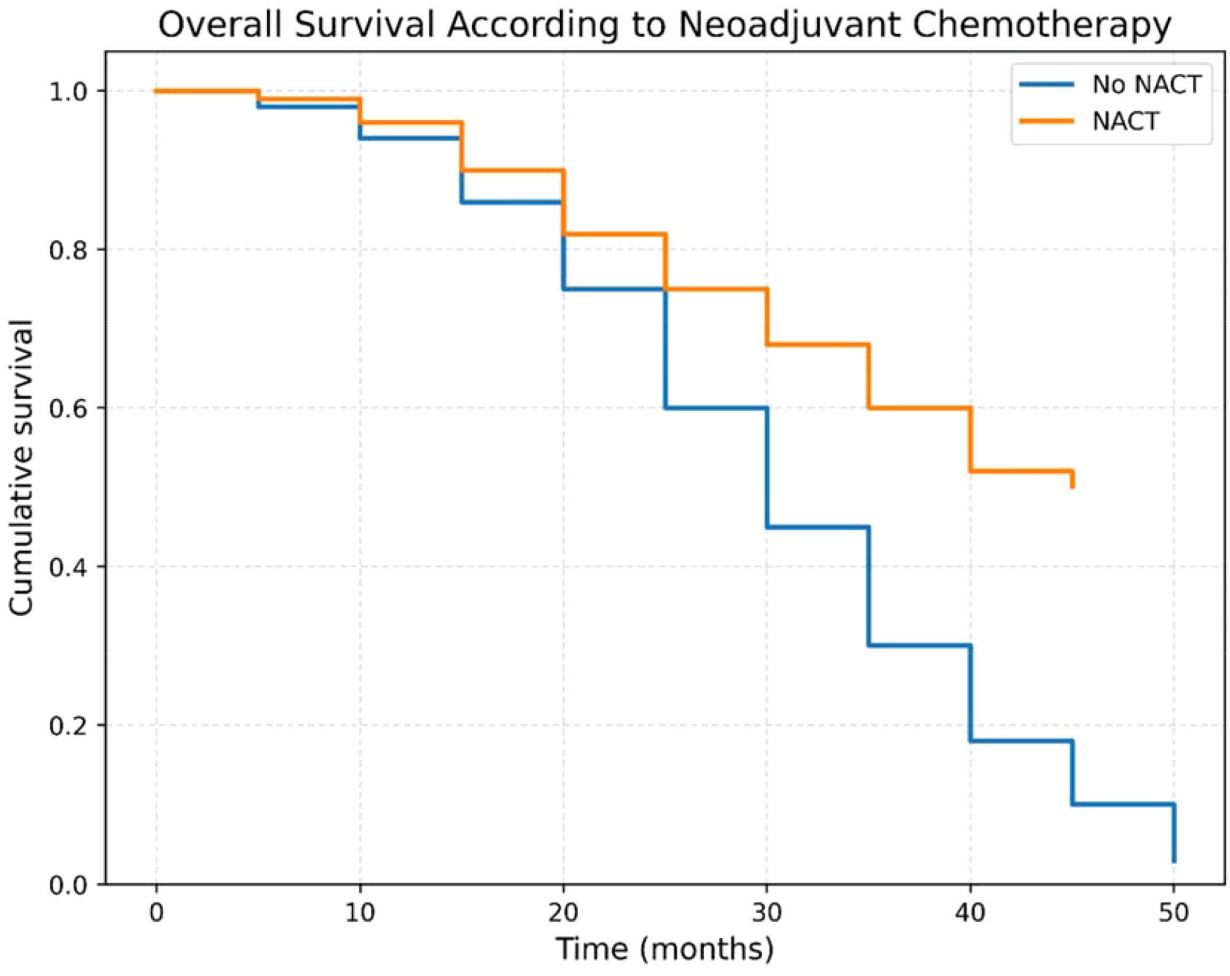
Overall survival curve of patients who underwent CRS + HIPEC with vs. without NACT.

The survival rate for patients who underwent neoadjuvant chemotherapy (NACT) was 50%, with a median survival duration of 36.9 ± 13.8 months. In contrast, patients who did not receive NACT had a survival rate of 3.2%, with a median survival duration of 28.26 ± 10.34 months (**Figure 2**).

### Impact of peritoneal cancer index on survival

**Figure 3:**
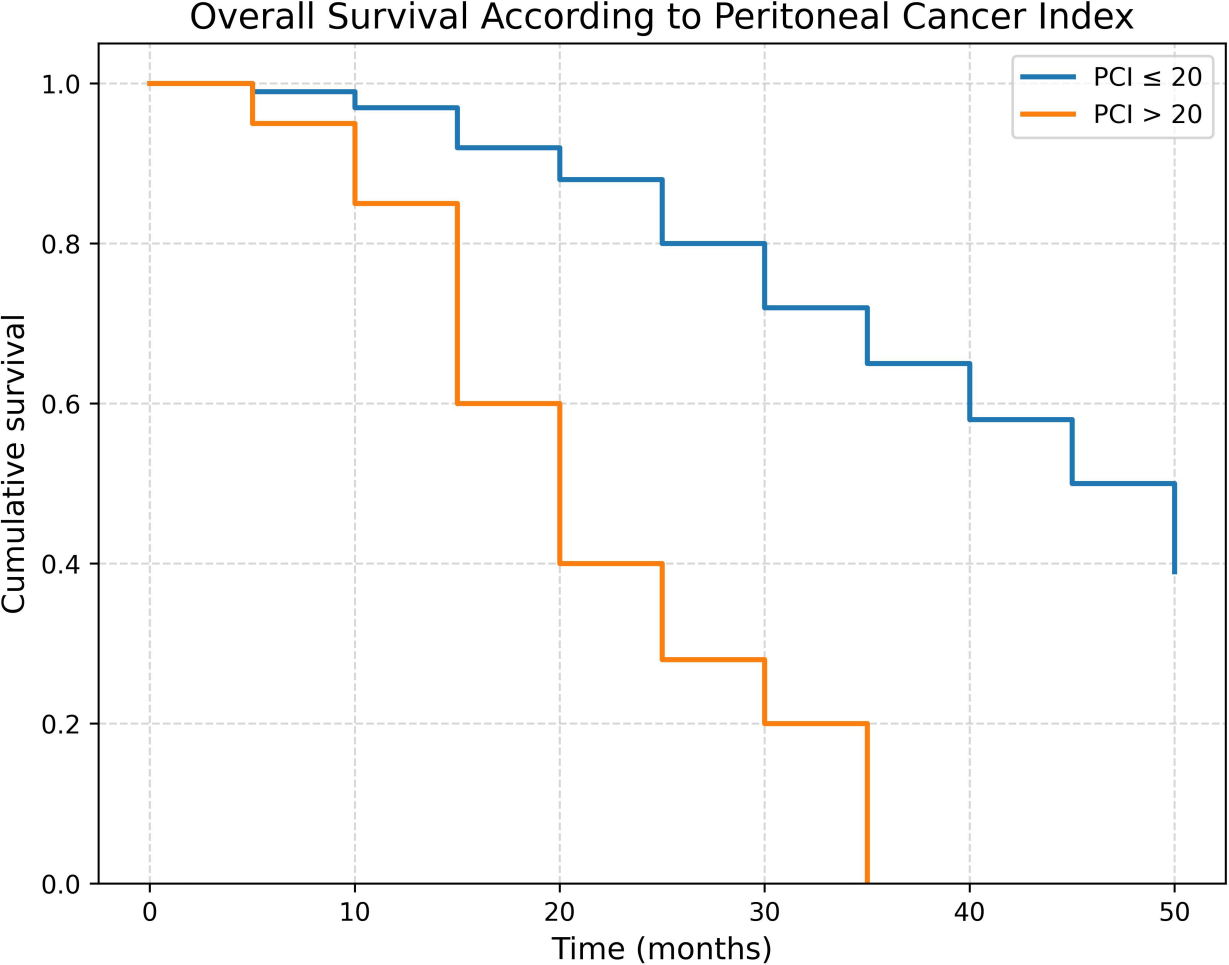
Overall survival curve in patients who underwent CRS + HIPEC, categorized by PCI scores (>20 vs. ≤20).

Among patients with a PCI score equal to or less than 20, the survival rate was 39%, with a median survival duration of 38.29 ± 10.49 months. In contrast, patients with a PCI score greater than 20 demonstrated a 0% survival rate, with a median survival duration of 20.65 ± 8.37 months (**Figure 3**).

### Impact of surgical technique on survival

**Figure 4:**
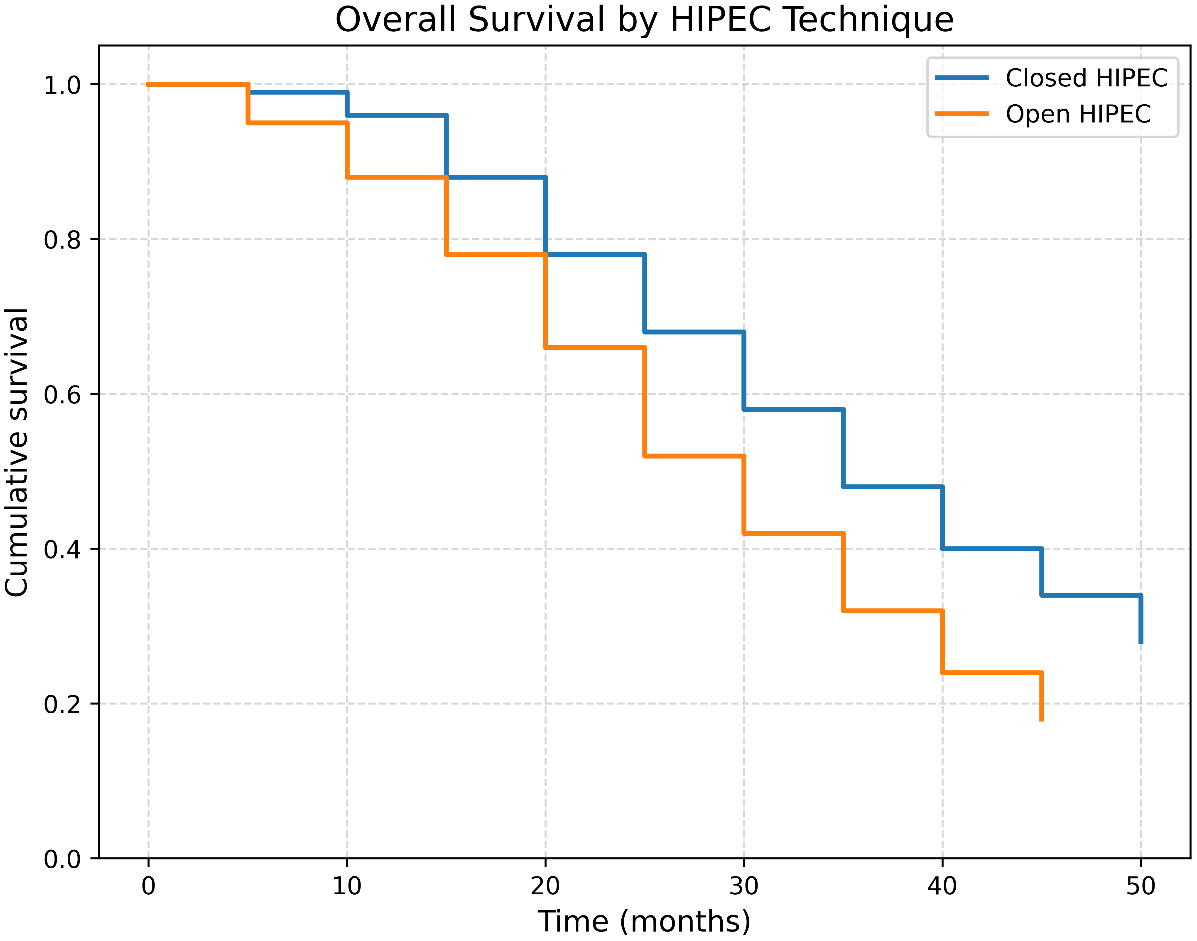
Overall survival curve in patients who underwent CRS + HIPEC, categorized by HIPEC technique (closed vs. open).

The survival rate among patients who underwent the closed HIPEC technique was 28.6%, with a median survival duration of 33.53 ± 12.54 months. Patients treated with the open technique demonstrated a survival rate of 16.7%, with a median survival duration of 28.33 ± 13.83 months. This difference was not statistically significant (**Figure 4**).

### Impact of completeness of cytoreduction on survival

**Figure 5:**
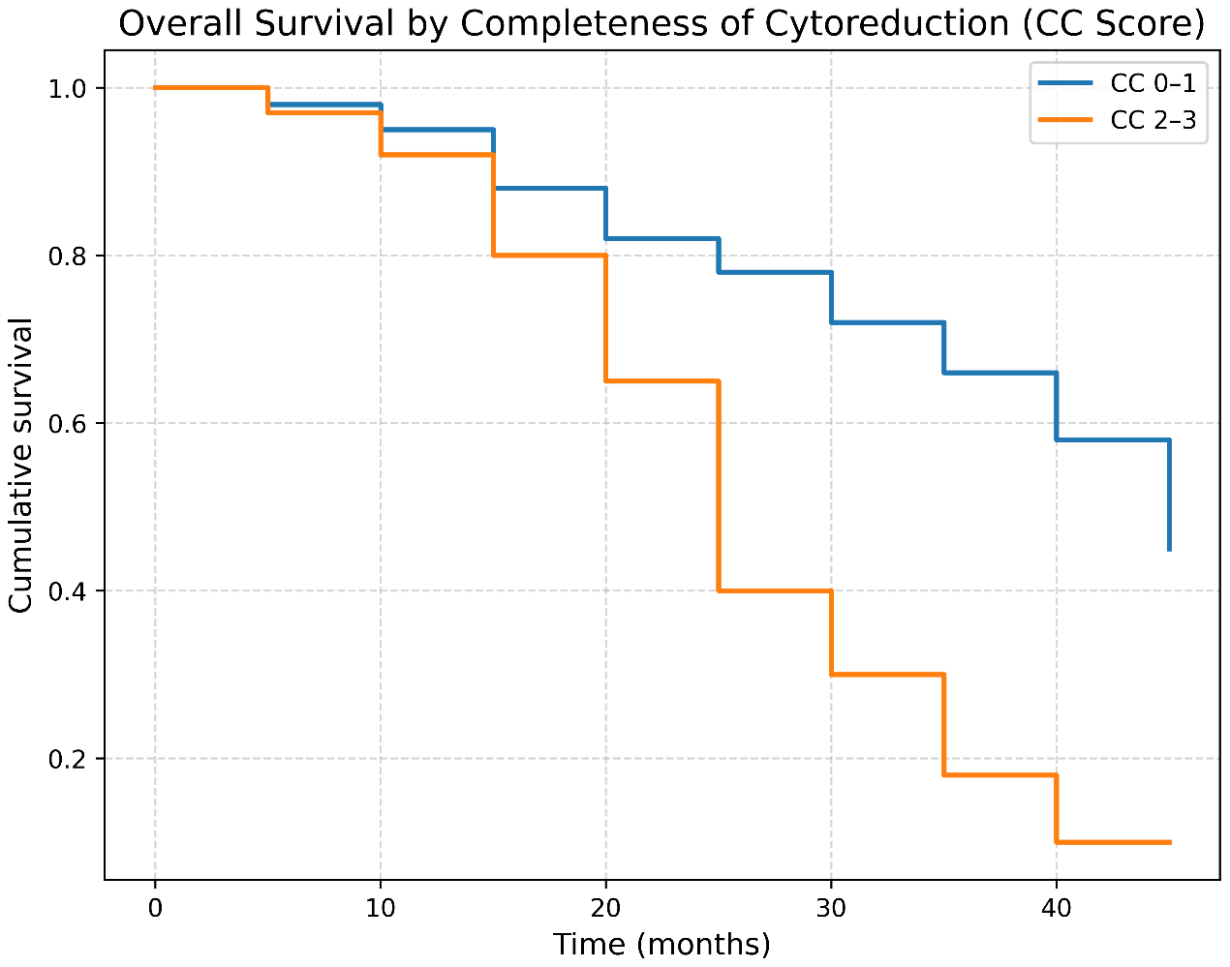
Overall survival curve in patients who underwent CRS + HIPEC, categorized by CC scores (0–1 vs. 2–3).

For patients with a CC score of 0–1, the survival rate was 44.8%, with a median survival duration of 38.41 ± 13.03 months. Conversely, patients with CC scores of 2–3 exhibited a markedly lower survival rate of 9.4%, with a median survival duration of 27.16 ± 10.22 months (**Figure 5**).

## Discussion

Peritoneal carcinomatosis has long been thought to represent an advanced stage of cancer best managed with palliative or supportive care. In recent decades, however, aggressive peritoneal therapies particularly cytoreductive surgery (CRS) combined with hyperthermic intraperitoneal chemotherapy (HIPEC) have resulted in substantial improvements in patient survival. The goal of these procedures is to prolong survival while maintaining an acceptable quality of life for patients with intra-abdominal malignancies (18, 19).

CRS is a complex surgical procedure involving extensive peritonectomy and resection of involved organs, with the primary aim of reducing macroscopic tumor burden. The advantages of additional HIPEC over systemic chemotherapy include direct exposure of residual peritoneal tumor cells to high concentrations of chemotherapeutic agents, improved tissue penetration, synergistic cytotoxic effects, and the independent antitumor effect of hyperthermia (18).

This study is particularly significant as it represents the first Iraqi experience with CRS and HIPEC and provides unique insights into the management of peritoneal carcinomatosis in a resource-limited and previously underreported setting. Historically, scientific and healthcare data from Iraq have been scarce due to political instability, prolonged conflict, and limited research infrastructure. As a result, patients are often present at more advanced stages of disease, which may partially explain the relatively high tumor burden observed in our cohort. The genetic diversity, environmental exposures, and socio-economic conditions in Iraq offer valuable contributions to the global understanding of oncologic disease patterns (20–22).

In our study, two patient groups were compared over a four-year follow-up period: one receiving neoadjuvant chemotherapy (NACT) followed by CRS+HIPEC and another undergoing CRS+HIPEC without NACT. A statistically significant survival advantage was observed in the NACT group, with a 50% four-year survival rate compared to only 3.2% in the non-NACT group (p = 0.0001). These findings are consistent with those reported by Zhou et al. (9) in colorectal cancer and further support the growing evidence that NACT plays a crucial role in patient selection, tumor downstaging, and optimization of surgical outcomes. Although most data supporting NACT originate from ovarian cancer literature, our results suggest its broader applicability (23, 24).

Regarding surgical technique, 80% of procedures in our cohort were performed using the closed HIPEC technique, while 20% utilized the open approach. This distribution is comparable to international practice patterns reported by Yoo et al. (25). Although no statistically significant difference in survival was observed between closed and open HIPEC techniques, this finding should be interpreted with caution due to the limited number of patients treated with the open approach (n = 12). The study was not powered to detect small or moderate differences between techniques, and therefore equivalence cannot be definitively established. However, the findings of Leiting et al. concluded that HIPEC technique alone does not independently influence long-term outcomes (26).

In our cohort, overall postoperative morbidity was approximately 33%, with major complications (Clavien–Dindo III–IV) occurring in 6% of patients and no perioperative mortality observed. These results compare favorably with published international data. Mehta et al. reported wide variability in CRS–HIPEC outcomes, with perioperative morbidity ranging from 30% to 70% and mortality from 0% to 18% (27). Similarly, Mohamed and Moran, in studies focusing on pseudomyxoma peritonei, reported procedure-related morbidity between 12% and 67.6% and mortality rates of 0% to 9%. Despite a high mean PCI (19.82) and the early institutional learning curve, the morbidity profile in our study lies at the lower end of these reported ranges, supporting the feasibility and acceptable safety of CRS–HIPEC in appropriately selected patients (28).

The mean PCI of 19.82 observed in our cohort is notably higher than that reported in many international series and warrants specific discussion. PCI is widely recognized as a key prognostic and predictive tool, with several centers considering PCI values above 15–20 as a relative or absolute contraindication to CRS, particularly in colorectal and gastric primaries. However, PCI thresholds are not universally standardized and should be interpreted in the context of tumor biology, institutional experience, and the likelihood of achieving complete cytoreduction.

A systematic review and meta-analysis by Narasimhan et al. (26) demonstrated that each incremental increase in PCI was associated with a 10% increase in mortality risk. Our findings align with this observation, as patients with PCI >20 experienced 100% mortality by approximately three years, whereas patients with PCI ≤20 demonstrated a four-year survival rate of 39% (p = 0.01). Importantly, CRS was selectively performed in high-PCI patients only when CC-0 or CC-1 resection was deemed achievable, reflecting a strategy that prioritizes cytoreduction completeness over PCI alone.

In addition to PCI, the Completeness of Cytoreduction (CC) score emerged as a decisive determinant of long-term survival. Patients with CC-0/CC-1 resections demonstrated a four-year survival rate of 44.8%, compared with only 9.4% among those with CC-2/CC-3 resections (p = 0.01). However, increasing evidence suggests that CC-1 should not be considered equivalent to CC-0, as even minimal residual disease may adversely affect long-term outcomes (29). In our cohort, descriptive analysis revealed differences between CC-0 and CC-1 subgroups, with survival rates of 33% (3/9 patients alive) and 50% (10/20 patients alive), respectively, while outcomes declined markedly for CC-2 (12.5%) and CC-3 (0%). Although the limited sample size, particularly in the CC-0 subgroup precluded statistically powered comparisons, these findings suggest that CC-1 may still confer inferior oncologic outcomes compared with complete macroscopic cytoreduction (CC-0), especially in patients with high PCI and advanced disease. Furthermore, the relatively high proportion of patients with CC ≥2 resections in our study likely reflects a combination of advanced disease at presentation, the tertiary referral nature of our center, and the early institutional learning curve associated with the first national CRS+HIPEC program in Iraq. As surgical experience and patient selection continue to evolve, improvements in cytoreduction completeness are anticipated, underscoring the importance of ongoing evaluation and refinement of selection criteria.

These findings reinforce existing evidence that complete cytoreduction remains the strongest prognostic factor in CRS and HIPEC, often outweighing the negative impact of a high PCI when optimal resection is achieved (30–32). Subsequently, incomplete cytoreduction is associated with a significantly worse survival outcome, emphasizing the critical role of both PCI and CC scores in predicting and influencing long-term patient prognosis (33–35).

This study has several limitations. First, its retrospective design introduces inherent selection and information bias. Second, it represents a single-center experience, which may limit generalizability. Third, the study population was histologically heterogeneous, reflecting real-world practice but limiting tumor-specific conclusions. Fourth, survival analyses were not stratified by primary tumor type or detailed histological subtype due to limited subgroup sizes. Fifth, the relatively small sample size, particularly within certain subgroups—limits statistical power and precludes definitive conclusions regarding technique-specific or subgroup comparisons. Finally, the influence of the institutional learning curve is particularly relevant, as this represents the first national CRS–HIPEC experience in Iraq, and outcomes may evolve with increasing experience and refined patient selection.

Continued research efforts in Iraq are essential to build on the significant contributions made by this study, with a focus on expanding insights into PC management. Future studies should explore refining surgical approaches, addressing the observed differences in survival outcomes, and assessing the impact of NACT more comprehensively. Collaborative efforts, both nationally and internationally, are crucial for pooling resources and expertise to further enhance the research landscape in Iraq. Additionally, integrating the findings of this study into clinical practice and healthcare policies will be instrumental in improving patient outcomes and establishing evidence-based guidelines for PC management.

## Conclusion

This study stands as the first from Iraq, offering valuable insights into the management of PC. The research revealed substantial differences in survival outcomes, emphasizing the critical impact of NACT. No statistically significant differences in outcomes were observed between closed and open HIPEC techniques; however, this observation is limited by the small number of patients in the open group and should not be interpreted as evidence of equivalence between techniques. Notably, patients with a higher PCI had significantly poorer outcomes, highlighting the importance of PCI as an important prognostic factor. The CC score further underscores the critical role of achieving optimal cytoreduction in influencing long-term survival. Ongoing research efforts, collaborative initiatives, and the integration of findings into clinical practice are recommended to enhance the understanding and management of PC.

## Data Availability

The original contributions presented in the study are included in the article/supplementary material. Further inquiries can be directed to the corresponding author.
